# Genetic variation spectrum in *ATP7B* gene identified in Latvian patients with Wilson disease

**DOI:** 10.1002/mgg3.297

**Published:** 2017-06-07

**Authors:** Agnese Zarina, Ieva Tolmane, Madara Kreile, Aleksandrs Chernushenko, Gunta Cernevska, Ieva Pukite, Ieva Micule, Zita Krumina, Astrida Krumina, Baiba Rozentale, Linda Piekuse

**Affiliations:** ^1^ Scientific Laboratory of Molecular Genetics Rīga Stradiņš University Riga Latvia; ^2^ Riga East Clinical University Hospital stationary “Latvian Centre of Infectious Diseases” Riga Latvia; ^3^ Faculty of Medicine University of Latvia Riga Latvia; ^4^ Children's Clinical University Hospital Riga Latvia

**Keywords:** *ATP7B* gene, copper metabolism, Wilson disease

## Abstract

**Background:**

Wilson disease (WD) is an autosomal recessive disorder of copper metabolism caused by allelic variants in *ATP7B* gene. More than 500 distinct variants have been reported, the most common WD causing allelic variant in the patients from Central, Eastern, and Northern Europe is H1069Q.

**Methods:**

All Latvian patients with clinically confirmed WD were screened for the most common mutation p.H1069Q by PCR Bi‐PASA method. Direct DNA sequencing of gene *ATP7B* (all 21 exons) was performed for the patients with WD symptoms, being either heterozygous for H1069Q or without it on any allele.

**Results:**

We identified 15 different allelic variants along with eight non‐disease‐causing allelic variants. Based on the gene molecular analysis and patients' clinical data variant p.His1069Gln was found in 66.9% of WD alleles. Wide clinical variability was observed among individuals with the same *ATP7B* genotype. The results of our study confirm that neurological manifestations of WD are typically present later than the liver disease but no significant association between the presence/absence of the most common genetic variant and mode of initial WD presentation or age at presentation was identified.

**Conclusions:**

(1) The most prevalent mutation in Latvian patients with Wilson disease was c.3207C>A (p.His1069Gln); (2) No significant phenotype–genotype correlation was found in Latvian patients with Wilson disease; (3) The estimated prevalence of Wilson disease in Latvia is 1 of 24,000 cases which is higher than frequently quoted prevalence of 1: 30,000.

## Introduction

Wilson disease (WD) is an autosomal recessive disorder of copper metabolism (OMIM #277900) caused by allelic variants in *ATP7B* gene (OMIM # 606882). The prevalence of WD is between 1:30,000 and 1:100,000 (Moller et al. 2011).

More than 500 distinct variants have been reported in the *ATP7B* gene. The most common in WD patients in Europe is H1069Q (traditional name by HGVS variant nomenclature NM_000053.3: c.3207C>A, NP_000044.2: p.His1069Gln, rs76151636). Its frequency varies – from 15% (in France) to 72% (in Poland) between WD patients (Gomes and Dedoussis 2016).

Clinical manifestations can vary widely, but the key features of WD are liver disease and cirrhosis, neuropsychiatric disturbances, Kayser–Fleischer rings in cornea, and acute episodes of hemolysis often in association with acute liver failure (European Association for Study of, 2012). A diagnostic scoring system was proposed at the 8th International Meeting on Wilson disease, Leipzig 2001 (Ferenci et al. 2003). The onset of WD is very variable, over 80% of patients have its symptoms within the first three decades of life (Gromadzka et al. 2005).

Definite genotype influence on a phenotype has not been established so far, although a number of studies have suggested potential relationships between the age of onset or type of manifestation and a specific genotype (Cocos et al. 2014).

In this study, we aimed to establish a relationship between the disparity in the clinical and biochemical features of WD in Latvian patients and presence/absence of allelic variant H1069Q in *ATP7B*.

## Material and Methods

From all subjects or their guardians the written informed consent for participation in this study was obtained. The study protocol was approved by the Latvian Central Medical Ethics Committee and the study was made according to the Declaration of Helsinki.

All patients included in the study had at least four points according to the WD scoring system (Ferenci et al. 2003 Jun). According to the initial symptoms, WD patients were categorized in the following groups as described elsewhere (Gromadzka et al. 2005 Dec): asymptomatic, hepatic, neurological/psychiatric, and neurological/hepatic.

Genomic DNA was extracted from peripheral blood by standard phenol/chloroform method (Sambrook and Russell 2006). Allelic variant c.3207C>A; p.H1069Q was tested by PCR‐Bi‐PASA (Polakova et al. 2007 Jun). The products of PCR Bi‐PASA method were visualized by 8% polyacrylamide gel electrophoresis.

Direct DNA sequencing of gene *ATP7B* (all 21 exons) was performed for the patients with WD symptoms, being either heterozygous for H1069Q or without it on any allele.

For the genetic variants nomenclature traditional names were used (if existing), or HGVS nomenclature according to reference sequences NG_008806.1, NM_000053.3 and NP_000044.2.

Allele and genotype frequencies were calculated by the direct count, the comparison between patients and a control group was done by Fisher exact test, accepting statistical significance if *P* < 0.05. Descriptive statistics was used for mean age, standard deviations. SPSS software v.22.0 (SPSS Inc., Chicago, IL, USA) was used to compare mean biochemical marker values between different patient groups (with different genotypes depending on the presence or absence of the most common mutation H1069Q). Parametric values were compared using ANOVA, and nonparametric data were evaluated with the Mann–Whitney test. Pathogenicity of genetic variants was ascertained using the WD allelic variant database: http://www.wilsondisease.med.ualberta.ca/database.asp, website http://www.ncbi.nlm.nih.gov/clinvar; for the unreported variants Polyphen‐2, SIFT, Mutation Taster, and Panther software prediction tools were used (Adzhubei et al. 2010), as well as both variants were tested in 93 healthy individuals in age more than 40 years, without any clinical signs of Wilson disease.

In the approximate birth prevalence detection of WD in Latvia, the previously reported approach was used (Reilly et al. 1993). The number of patients (symptomatic and asymptomatic siblings detected in family screening) with WD was divided by the total number of births (the data were obtained from the Latvian Central Statistical Bureau) in the period of time from 1964 to 2012. Patients born before 1964 were excluded from prevalence estimation since biochemical diagnostics of WD in Latvia are available from 1980, and 16 years were taken before – since it is the average age for WD expression (Coffey et al. 2013).

## Results

In total, 23 different genetic variants in gene *ATP7B* were found. Thirteen variants previously described as the disease causing variants (WD mutation database: http://www.wilsondisease.med.ualberta.ca/database.asp) were identified. Two variants identified in this study were novel: c.3800A>G (p.Asp1267Gly) in exon 18 and c.3971A>C (p.Asn1324Thr) in exon 19. For pathogenicity analysis of the identified variants four prediction tools (Polyphen‐2, SIFT, Mutation Taster, and Panther software) showed that both variants are probably damaging or disease causing variants (see Table S2). The novel variant in exon 18 was found in one allele of control group (the minor allele frequency – 0.005), showing no statistically significant difference with frequency in the patient group (*P* > 0.05). The variant in exon 19 was not found in any allele of healthy control.

**Figure 1:**
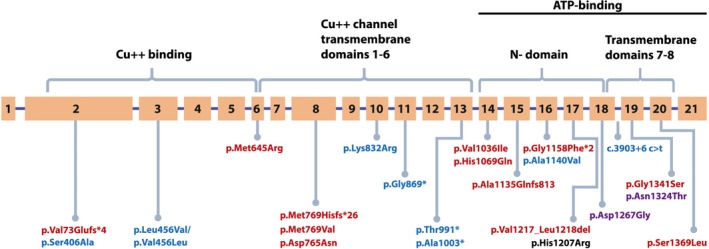
Variants in gene *ATP7B* found in Latvian patients with Wilson disease. Benign variants are shown in blue, disease‐causing variants – in red, but variants with uncertain clinical significance – in purple.

The rate of disease‐causing variant detection in this study was 82.35% (Fig. 1): the most prevalent was c.3207C>A (p.His1069Gln), found in 66.9% of alleles followed by c.2304dupC (p.Met769Hisfs*26) – in 3.7% of alleles. Most of the other variants were detected in a very small percentage (in one individual/family). Eight previously reported (Figus et al. 1995; Thomas et al. 1995; Shah et al. 1997; Cox et al. 2005) benign variants were identified (Fig. 1).

A total of 68 individuals (34 males and 34 females, aged between three and 48 years (mean age ± SD, 22.76 ± 10.32 years)) of 60 unrelated families were included in the study. All of the eight siblings were asymptomatic at diagnosis. Thirty‐two of 60 (53%) patients presented with hepatic symptoms, 14 (23%) with neurological, and 14 (23%) with both hepatic and neurological. The first symptoms in patients with hepatic presentation appeared at earlier age (mean age 18.69 ± 8.85 years) compared to patients with neurological or mixed presentation (mean age 26.38 ± 10.28 years; *P* = 0.02). The mean age of patients (asymptomatic patients were excluded) at initial symptoms of WD was 23.17 ± 10.65 years (median: 23.5 years, range: 3–48 years). More detailed characteristics of the WD patients can be found in the Table S1.

The segregation of clinical symptoms and the age of onset according to the genotype in Latvian WD patients (asymptomatic relatives are excluded) is shown in Table 1.

**Table 1:** Genotypes and phenotypes of patients with WD (without asymptomatic relatives) in Latvia

	*ATP7B* genotype		
	H1069Q homozygotes	Compound heterozygous with H1069Q mutation	Other genotypes
Total number of patients	32 (53%)	15 (25%)	13 (22%)
Neurological manifestation (%)	5 (7%)	2 (3%)	7 (12%)
Hepatic manifestation (%)	18 (30%)	11 (18%)	3 (5%)
Neurological/hepatic manifestation (%)	9 (15%)	2 (3%)	3 (5%)
Mean age at manifestation (years ± SD)	24.28 ± 10.7	16.87 ± 8.41	27.39 ± 10.2
Median age at manifestation (range)	24 (3–48)	15 (5–30)	29 (11–47)

The final number of patients enrolled in the analyses of phenotype–genotype correlation was 60 (eight asymptomatic relatives were excluded). Upon comparing *ATP7B* genotype with clinical appearance and age at presentation of Wilson disease, no statistically significant changes were found.

In total 53 patients and eight relatives were included in the birth prevalence estimation study. The birth prevalence in the whole period was 4.17 cases per 100,000 children born alive or 1 of 23,980.

## Discussion

The most common WD causing allelic variant in Europe is H1069Q. In Latvia it was found in 66.9%, which matches to its frequency in most of European populations (Gomes and Dedoussis 2016). Only three variants in addition to the most common were detected in more than one allele which suggests of a wide genetic heterogeneity in Latvian patients with WD. Variants in exons 14 and 8 were found in 72.8% of alleles indicating that exons 8 and 14 are two very important regions for detecting genetic changes in Latvian patients with WD.

Most of the variants found in our study were reported before. Variant c.3472_3482del; p.Gly1158Phe*2 has been reported only in two other neighboring populations – Polish (Gromadzka et al. 2005) and Lithuanians (Kucinskas et al. 2008), suggesting that there could be a common ancestor in all three populations. Variant c.3620A>G (p.His1207Arg) has conflicting interpretation of pathogenicity in literature as it has been described as benign (Loudianos et al. 1999) and pathogenic (Abdelghaffar et al. 2008). Because of the conflicting results, it is planned to do further sequencing for the patient including promoter region, 5′UTR and 3′UTR regions. Two variants identified in this study were novel with uncertain clinical significance: c.3800A>G (p.Asp1267Gly) and c.3971A>C (p.Asn1324Thr). Previously reported variants of the first variant's particular position c.3800A>C (Okada et al. 2000) and c.3800A>T (Davies et al. 2008) and its location at metal‐binding domain (www.uniprotkb.org) indicate a high probability of pathogenicity. Four prediction tools showed that both novel variants are probably damaging ones. Variant c.3971A>C (p.Asn1324Thr) was not found in any allele of healthy controls, indirectly confirming the high pathogenicity of it. Variant c.3800A>G (p.Asp1267Gly) was found in one allele of healthy controls and the frequency of it did not differ from the patient group, but as WD is an autosomal recessive disease, disease causing variants can be found in healthy individuals in a heterozygotic state, but nevertheless further investigations are needed for the confirmation.

The rate of disease‐causing variant detection in this study was 82.35%, but 17.65% of alleles remained unidentified. It can be explained by different causes – either the remaining variants are located in noncoding regions of the gene *ATP7B* or WD can be also caused by large deletions, as well as WD can have unusual mutational mechanisms (e.g., WD can be caused by mutations in other genes besides the gene *ATP7B*).

The previous studies devoted to possible genotype–phenotype correlation have reported contradictory conclusions (Stapelbroek et al. 2004; Vrabelova et al. 2005; Chappuis et al. 2007; Bem et al. 2013). In meta‐analysis based on the results from 11 different centers, H1069Q was associated with late and mainly neurological presentation of WD (Stapelbroek et al. 2004). However, in some other studies no significant correlation was found between H1069Q presence/absence and clinical presentation of WD (Vrabelova et al. 2005; Chappuis et al. 2007; Bem et al. 2013). The results of our study confirm that neurological manifestations of WD typically appear later than the liver disease, as indicated previously (Stapelbroek et al. 2004); however, no significant association between the presence/absence of the most common genetic variant and mode of initial WD presentation or age at presentation was identified.

In our study, the prevalence of Wilson disease in Latvia is approximately 4.17 cases per 100,000 (or 1: ~24,000), which is higher than the frequently quoted prevalence of 1: 30,000 (Coffey et al. 2013). The birth prevalence is an approximate number, in light of the fact that some genotypically confirmed individuals remained asymptomatic and undetected by the end of the study ascertainment period, which is just an indication toward the fact that the diagnostics of WD is very accurate and well provided in Latvia. In order to prove the last statement different methods of prevalence detection should be applied.

## Conflict of Interest

Authors declare no conflict of interest.

## Supporting information


**Table S1.** Characteristics of WD patients in Latvia.

**Table S2.** Results of pathogenicity prediction for two novel variants.
